# Reinforcement Q-Learning-Based Adaptive Encryption Model for Cyberthreat Mitigation in Wireless Sensor Networks

**DOI:** 10.3390/s25072056

**Published:** 2025-03-26

**Authors:** Sreeja Balachandran Nair Premakumari, Gopikrishnan Sundaram, Marco Rivera, Patrick Wheeler, Ricardo E. Pérez Guzmán

**Affiliations:** 1 Department of Information Technology, Karpagam College of Engineering, Myleripalayam Village, Coimbatore 641032, Tamil Nadu, India; sreejabp@gmail.com; 2 School of Computer Science and Engineering, VIT-AP University, Amaravati 522241, Andhra Pradesh, India; gopikrishnan.s@vitap.ac.in; 3 Department of Electrical and Electronic Engineering, Faculty of Engineering, University of Nottingham, Nottingham NG7 2GT, UK; pat.wheeler@nottingham.ac.uk; 4 Department of Computer Science, Faculty of Engineering, Universidad de Talca, Curicó 3460000, Chile; riperez@utalca.cl

**Keywords:** adaptive encryption, energy efficiency, Q-learning, real-time threat detection, reinforcement learning, resource-constrained networks, wireless sensor networks, security optimization

## Abstract

The increasing prevalence of cyber threats in wireless sensor networks (WSNs) necessitates adaptive and efficient security mechanisms to ensure robust data transmission while addressing resource constraints. This paper proposes a reinforcement learning-based adaptive encryption framework that dynamically scales encryption levels based on real-time network conditions and threat classification. The proposed model leverages a deep learning-based anomaly detection system to classify network states into low, moderate, or high threat levels, which guides encryption policy selection. The framework integrates dynamic Q-learning for optimizing energy efficiency in low-threat conditions and double Q-learning for robust security adaptation in high-threat environments. A Hybrid Policy Derivation Algorithm is introduced to balance encryption complexity and computational overhead by dynamically switching between these learning models. The proposed system is formulated as a Markov Decision Process (MDP), where encryption level selection is driven by a reward function that optimizes the trade-off between energy efficiency and security robustness. The adaptive learning strategy employs an ϵ-greedy exploration-exploitation mechanism with an exponential decay rate to enhance convergence in dynamic WSN environments. The model also incorporates a dynamic hyperparameter tuning mechanism that optimally adjusts learning rates and exploration parameters based on real-time network feedback. Experimental evaluations conducted in a simulated WSN environment demonstrate the effectiveness of the proposed framework, achieving a 30.5% reduction in energy consumption, a 92.5% packet delivery ratio (PDR), and a 94% mitigation efficiency against multiple cyberattack scenarios, including DDoS, black-hole, and data injection attacks. Additionally, the framework reduces latency by 37% compared to conventional encryption techniques, ensuring minimal communication delays. These results highlight the scalability and adaptability of reinforcement learning-driven adaptive encryption in resource-constrained networks, paving the way for real-world deployment in next-generation IoT and WSN applications.

## 1. Introduction

The rapid expansion of 6G communication networks and proliferation of the Internet of Things (IoT) have led to a surge of data-driven applications that require real-time, intelligent, and secure communication. Wireless Sensor Networks (WSNs) play a crucial role in smart environments, healthcare, industrial automation, disaster management, and critical infrastructure monitoring. However, as WSNs become an integral part of 6G-driven IoT ecosystems, they face new challenges, particularly in terms of resilience to cyberattacks, resource constraints, and dynamic security threats.

WSNs are widely deployed in various critical domains, including smart cities, healthcare, industrial automation, agriculture, disaster management, and military surveillance. In smart cities, WSNs are used for traffic monitoring, pollution control, and smart grids, providing real-time data for efficient urban planning. In healthcare and remote patient monitoring, WSNs enable continuous patient tracking through wearable devices, ensuring real-time health analytics and emergency alerts. In industrial automation, WSNs support predictive maintenance, process control, and environmental sensing for Industry 4.0 and IIoT applications. In agriculture, they help detect soil moisture, analyze crop health, and monitor climate, improving productivity. WSNs also play a crucial role in disaster management by enabling early detection of natural calamities like earthquakes and floods, facilitating rapid response and risk mitigation. Additionally, in military and surveillance applications, they are utilized for battlefield monitoring, intrusion detection, and border security, strengthening tactical operations.

Despite their extensive applications, WSNs face several limitations that hinder their integration into 6G and advanced IoT ecosystems, particularly in the context of cybersecurity, adaptability, and energy efficiency. Traditional WSN security approaches rely on static encryption methods such as AES, RSA, and ECC, which lack adaptability to dynamic cyber threats. These methods do not dynamically adjust the strength of the encryption based on network conditions, making them vulnerable to evolving attacks. Furthermore, WSNs operate in open and decentralized environments, making them highly prone to cyberattacks such as DDoS, black-hole, wormhole, and data injection attacks. Traditional intrusion detection systems (IDSs) are ineffective in real-time threat mitigation, leading to delayed responses and potential data breaches.

Energy constraints are another significant limitation in resource-limited WSN nodes. These nodes are battery-powered, which restricts the implementation of computationally intensive security mechanisms. Deploying robust encryption without energy optimization leads to rapid battery depletion and reduced network life. In addition, 6G networks require low-latency adaptive security mechanisms to ensure real-time protection for time-sensitive applications. However, traditional security models fail to balance security strength with latency and energy efficiency, resulting in either excessive computational overhead or weak security implementations. Furthermore, many existing WSN security models are rule-based or machine learning-driven, but they lack continuous learning capabilities. This makes them ineffective against zero-day attacks or adversarial manipulations, leading to compromised network integrity.

### 1.1. Security Issues in WSN

The evolving threat landscape in 6G-driven WSN deployments demands a shift from conventional security paradigms to more adaptive and autonomous approaches. Scalability remains a challenge, as traditional security frameworks struggle to accommodate large-scale WSN deployments without significantly increasing computational overhead. Furthermore, the lack of standardized security models results in fragmented security strategies, making it difficult to develop a unified security framework that is applicable to various WSN applications. A significant trade-off also exists between security and performance, as stronger security mechanisms often increase computational complexity, negatively affecting latency, energy efficiency, and real-time responsiveness.

These challenges highlight the urgent need for advanced security solutions in WSNs to meet the demands of 6G-driven IoT ecosystems. Future security frameworks must address cyberattack mitigation, resource optimization, and real-time adaptability while ensuring minimal impact on network performance. As WSNs continue to evolve, integrating intelligent, adaptive security mechanisms will be crucial to maintaining network resilience and enabling secure communication in next-generation IoT environments.

Wireless Sensor Networks (WSNs) have revolutionized modern technology by enabling efficient data collection, transmission, and processing across a wide range of applications. These networks consist of distributed sensor nodes equipped with sensing, computation, and communication capabilities, making them integral to areas such as environmental monitoring, healthcare, military operations, smart agriculture, and industrial automation [1,2]. The ability of WSNs to operate in resource-constrained and often inaccessible environments has cemented their importance in modern technological ecosystems.

Despite their advantages, WSNs face significant challenges due to their inherent limitations, including constrained energy resources, computational power, and storage capacities [3]. These limitations make WSNs particularly vulnerable to various cyber threats, ranging from data injection and distributed denial-of-service (DDoS) attacks to black-hole and wormhole attacks [4,5]. Cyberattacks not only compromise data confidentiality and integrity, but also disrupt network functionality, jeopardizing critical applications such as disaster management and military surveillance. The need for robust security mechanisms to protect WSNs from these threats has become increasingly critical [6].

Existing security mechanisms, including traditional encryption techniques such as AES, RSA, and ECC, rely on static security levels that do not adapt to evolving threats. Although lightweight encryption techniques such as LBC, Hybrid AES-RSA, and DTE optimize computational efficiency to some extent, they still rely on predefined security thresholds rather than dynamically adjusting to real-time cyber threats. Furthermore, machine learning-based security models improve threat detection, but lack adaptive mechanisms that dynamically adjust security policies. These limitations highlight the urgent need for an advanced security framework capable of dynamically adapting encryption levels based on real-time threat intelligence.

One of the critical challenges in traditional security mechanisms is their high energy consumption, which significantly impacts the efficiency and longevity of WSNs. Standard encryption techniques require substantial computational resources, leading to increased power consumption. Although lightweight encryption approaches moderately optimize energy usage, they do not explicitly balance security and energy efficiency. Machine learning-based models focus on threat detection but often overlook energy optimization, leading to inefficient security implementations in WSNs.

The existing encryption techniques [5,7,8,9,10] in WSNs impose high computational overhead or lack adaptability to evolving cyber threats. Static encryption methods fail to optimize security and energy efficiency simultaneously, making resource-constrained WSNs highly vulnerable to attacks such as distributed denial-of-service (DDoS), black-hole, and data injection attacks. To address this gap, there is a need for a reinforcement learning-based adaptive encryption framework that dynamically adjusts encryption levels based on real-time network conditions, ensuring robust security while optimizing energy consumption. Mitigating cyberattacks in WSNs requires adaptive and resource-efficient solutions that can respond dynamically to evolving threats. Traditional cryptographic techniques, including AES and RSA, provide strong security guarantees but are computationally intensive, making them unsuitable for energy-constrained WSN environments [11,12]. Lightweight encryption techniques, such as elliptic curve cryptography (ECC), have emerged as viable alternatives due to their smaller key size and reduced energy consumption [13]. However, static encryption methods often lack the flexibility to adapt to real-time network conditions and evolving threat landscapes [14].

The threat detection and classification mechanisms in existing approaches are often inadequate, further increasing the vulnerability of WSNs. Traditional encryption mechanisms do not provide an inherent threat classification, making them ineffective in mitigating targeted cyber threats. Lightweight encryption techniques incorporate basic anomaly detection, but these methods lack the sophistication required to address complex attack scenarios. Although machine learning-based approaches enhance threat detection accuracy, they still rely on pre-trained datasets, which limits their ability to respond to novel or evolving threats in real time.

Cyberattack resilience is another area where traditional security models fall short. Traditional encryption schemes are static and lack the ability to respond to dynamically evolving attack strategies. Lightweight encryption techniques provide moderate resilience, but rely on threshold-based security, which is ineffective against sophisticated cyber threats. Machine learning-based models detect attacks efficiently, but lack a proactive mitigation mechanism.

### 1.2. Motivation

The motivation for this research is driven by the growing complexity of cyber threats targeting WSNs, including distributed denial-of-service (DDoS), black-hole, wormhole, and data injection attacks. Existing machine learning-based anomaly detection systems exhibit limitations in real-time adaptation and computational efficiency, making them unsuitable for resource-constrained WSN environments. Additionally, traditional cryptographic mechanisms impose high energy overheads, which significantly impact network longevity. A major limitation of existing approaches is their inability to balance security and latency. Traditional encryption methods prioritize security, but at the cost of increased latency, which negatively impacts network performance. Although lightweight encryption techniques reduce latency, they often compromise security effectiveness. Machine learning models improve security, but do not effectively optimize latency. Existing security models also lack comprehensive evaluation frameworks. Traditional encryption mechanisms assess only basic security effectiveness without considering energy efficiency or system performance. Lightweight encryption models evaluate security–energy trade-offs, but fail to measure network-wide performance, including latency and mitigation efficiency. Machine learning-based models primarily assess detection accuracy, but do not incorporate real-time performance considerations.

Adaptive encryption, which dynamically adjusts security parameters based on real-time feedback, has gained significant attention as a means to optimize the trade-off between security and resource efficiency in WSNs [15]. Reinforcement learning (RL), a branch of machine learning, has shown particular promise in enabling such adaptability. Using RL techniques, WSNs can make intelligent decisions to dynamically scale encryption levels, balancing energy consumption, latency, and security [1]. Among RL methods, Q-learning stands out for its ability to model and solve sequential decision-making problems in resource-constrained environments. It enables nodes to learn optimal policies for setting encryption levels by interacting with the environment and receiving feedback in the form of rewards [5].

The integration of Q-learning into adaptive encryption frameworks has demonstrated significant potential to improve the resilience of WSNs to cyberattacks [11]. Q-learning allows nodes to dynamically adjust encryption levels in response to network conditions, ensuring optimal performance across key metrics such as packet delivery ratio (PDR), latency, and energy efficiency [3,11]. Furthermore, the combination of Q-learning with other advanced techniques, such as blockchain for secure communication and anomaly detection for threat identification, provides a comprehensive approach to mitigate cyber threats in distributed WSNs [13].

### 1.3. Research Contributions

This research introduces a reinforcement learning-based adaptive encryption framework to mitigate cyberattacks in wireless sensor networks. The proposed model integrates Q-learning with advanced encryption scaling techniques to dynamically adjust encryption levels based on real-time threat detection while minimizing energy consumption. The key contributions of this research are the following:

- Development of a reinforcement learning-driven adaptive encryption framework that utilizes Q-learning-based policy optimization to select encryption levels based on network threat conditions. The model incorporates a Markov Decision Process (MDP) formulation to define state transitions and reward functions for optimizing energy efficiency and security robustness [16].
- Integration of dynamic Q-learning (Algorithm 1) for energy-efficient encryption scaling in low-threat conditions. The algorithm dynamically adjusts encryption complexity by evaluating the energy cost–security trade-off, ensuring resource conservation while maintaining sufficient security levels. It employs an ϵ-greedy exploration–exploitation strategy with an adaptive decay mechanism to improve learning convergence in dynamic WSN environments.
- Implementation of Double Q-Learning (Algorithm 2) for robust security adaptation under high-threat scenarios. The use of dual Q-value function approximation reduces the overestimation bias inherent in traditional Q-learning. By maintaining two separate Q-value estimations and alternating updates, the framework enhances security decision-making, effectively mitigating advanced cyberattacks such as DDoS, black-hole, and data injection attacks.
- Design of a Hybrid Policy Derivation Algorithm (Algorithm 3) that optimally balances encryption levels by combining the strengths of dynamic Q-learning and double Q-learning. The hybrid policy integrates real-time threat assessment using a feedforward neural network-based anomaly detection model (Algorithm 4) to ensure adaptive encryption decision-making without excessive computational overhead.
- Integration of a deep learning-based anomaly detection system (Algorithm 4) for real-time threat classification using packet delivery ratio (PDR), latency, and anomaly scores. The model utilizes a feedforward neural network trained on historical network traffic to classify low-, moderate-, and high-threat states, guiding the encryption adaptation process.
- Introduction of a dynamic hyperparameter tuning mechanism for reinforcement learning updates, ensuring adaptive learning rate adjustment based on network conditions. This optimizes Q-learning convergence speed, enhancing the model’s adaptability to dynamic WSN environments.
- Extensive evaluation in a simulated wireless sensor network environment, demonstrating a 30.5% reduction in energy consumption, a 92.5% packet delivery ratio, and a 37% reduction in transmission latency.
- Enhanced security effectiveness with a 94% attack mitigation efficiency against DDoS, black-hole, and data injection attacks.
- Practical applicability and future scalability considerations, including integration with blockchain-based encryption key management for decentralized security enhancement, deployment in low-power IoT hardware such as ARM Cortex and Raspberry Pi for real-world energy profiling, and extension to multi-agent reinforcement learning for decentralized encryption decision-making.

**Algorithm 1** Enhanced Q-Learning with Dynamic Parameter Adjustment for Adaptive Encryption.1:Initialize Q-values Q(s,a) arbitrarily for all state-action pairs (s,a)2:Initialize learning rate α, discount factor γ, and exploration rate ϵ3:Define maximum and minimum exploration rates $\epsilon_{\max}$, $\epsilon_{\min}$, and decay rate λ4:**for** each episode **do**5:      Initialize state *s*6:      **for** each time step **do**7:            Select action *a* using ϵ-greedy policy based on Q(s,a)8:            Execute action *a*, observe reward *r*, and next state $s^{\prime }$9:            Update Q-value using$$Q\left(s,a\right)\leftarrow Q\left(s,a\right)+\alpha \left[r+\gamma \max_{a^{\prime }}Q\left(s^{\prime },a^{\prime }\right)-Q\left(s,a\right)\right]$$10:           Update state $s\leftarrow s^{\prime }$11:      **end for**12:      Decay exploration rate $\epsilon \leftarrow \epsilon_{\min}+\left(\epsilon_{\max}-\epsilon_{\min}\right)e^{-\lambda \cdot \mathrm{episode}}$13:**end for**14:**return** Optimized Q-values Q(s,a)

**Algorithm 2** Double Q-Learning for Adaptive Encryption Scaling.
1:Initialize Q-tables $Q_{1}\left(s,a\right)$ and $Q_{2}\left(s,a\right)$ arbitrarily for all state–action pairs (s,a)2:Initialize learning rate α, discount factor γ, and exploration rate ϵ3:**for** each episode **do**4:      Initialize state *s*5:      **for** each time step **do**6:            Select action *a* using ϵ-greedy policy:$$a=\arg\max_{a}\left[Q_{1}\left(s,a\right)+Q_{2}\left(s,a\right)\right]$$7:            Execute action *a*, observe reward *r*, and next state $s^{\prime }$8:            Update Q-tables using$$\;\;\;Q_{1}\left(s,a\right)\leftarrow Q_{1}\left(s,a\right)+\alpha \left[r+\gamma Q_{2}\left(s^{\prime },\arg\max_{a^{\prime }}Q_{1}\left(s^{\prime },a^{\prime }\right)\right)-Q_{1}\left(s,a\right)\right]$$$$\;\;\;Q_{2}\left(s,a\right)\leftarrow Q_{2}\left(s,a\right)+\alpha \left[r+\gamma Q_{1}\left(s^{\prime },\arg\max_{a^{\prime }}Q_{2}\left(s^{\prime },a^{\prime }\right)\right)-Q_{2}\left(s,a\right)\right]$$9:             Update state $s\leftarrow s^{\prime }$10:      **end for**11:      Decay exploration rate $\epsilon \leftarrow \epsilon_{\min}+\left(\epsilon_{\max}-\epsilon_{\min}\right)e^{-\lambda \cdot \mathrm{episode}}$12:
**end for**
13:**return** Optimized Q-tables $Q_{1}\left(s,a\right)$ and $Q_{2}\left(s,a\right)$


**Algorithm 3** Adaptive Cyberattack Mitigation with Hybrid Encryption.
1:Initialize global parameters: learning rate α, discount factor γ, exploration rate ϵ, and decay rate λ2:Initialize two Q-tables $Q_{1}\left(s,a\right)$ and $Q_{2}\left(s,a\right)$ for adaptive policy learning3:**for** each episode **do**4:      Initialize state *s* based on current network conditions (e.g., threat levels, energy metrics)5:      **while** episode is not terminated **do**6:            Detect current threat level in the network7:            **if** threat level is high **then**8:                  **Call Algorithm 1: Double Q-Learning** for secure encryption scaling9:                  Update $Q_{1}$ and $Q_{2}$ based on Algorithm 2’s policy10:           **else**11:                 **Call Algorithm 4: Dynamic Q-Learning** for energy-efficient encryption scaling12:                 Update $Q_{1}$ and $Q_{2}$ based on Algorithm 1’s policy13:           **end if**14:           Observe reward *r* and next state $s^{\prime }$ from the environment15:           Update state $s\leftarrow s^{\prime }$16:      **end while**17:      Decay exploration rate ϵ using$$\epsilon \leftarrow \epsilon_{\min}+\left(\epsilon_{\max}-\epsilon_{\min}\right)e^{-\lambda \cdot \mathrm{episode}}$$18:
**end for**
19:Derive final hybrid policy π(s) using$$\pi \left(s\right)=\arg\max_{a}\left[Q_{1}\left(s,a\right)+Q_{2}\left(s,a\right)\right]$$20:**return** Optimized Q-tables $Q_{1}$, $Q_{2}$, and hybrid policy π(s)


**Algorithm 4** Deep Learning-Based Anomaly Detection.
1:Load the trained feedforward neural network model M2:Initialize threshold values $\tau_{\mathrm{low}}$ and $\tau_{\mathrm{high}}$ for anomaly scores3:Input real-time network metrics: X={PDR,Latency,PacketDropRate}4:Compute the anomaly score A=M(X) using the neural network model5:
**if** $A\leq \tau_{\mathrm{low}}$ **then**
6:      Classify as low threat7:      Trigger energy-efficient actions (Algorithm 1)8:
**else if** $A>\tau_{\mathrm{high}}$ **then**
9:      Classify as high threat10:     Trigger robust encryption scaling (Algorithm 2)11:
**else**
12:     Classify as moderate threat13:     Balance actions between energy efficiency and security14:
**end if**
15:Return threat classification and initiate appropriate countermeasures


The experimental results validate that the proposed model effectively mitigates cyber threats while maintaining optimal resource utilization. Using reinforcement learning for encryption adaptation, the framework ensures robust security without imposing excessive computational overhead. The ability to dynamically adjust encryption strength based on real-time network conditions makes this approach particularly suitable for resource-constrained environments such as wireless sensor networks and Internet of Things applications. The results demonstrate that the proposed method achieves a balanced trade-off between security, energy efficiency, and latency, making it a viable solution for secure real-time communication in dynamic network environments. The rest of this paper is structured as follows. Section 2 reviews existing approaches to WSN security, emphasizing lightweight and adaptive techniques. Section 3 details the methodology of the proposed adaptive encryption framework. Section 4 presents experimental evaluations and results, and Section 5 concludes with insights and potential future research directions.

## 2. Literature Review

The security of WSNs and IoT networks has emerged as a significant research focus, driven by the imperative to counter cyber threats and ensure reliable network functionality. In the realm of IoT security, ref. [17], introduced a blockchain-based encryption method with the generation of dynamic spider web keys, achieving reduced latency and improved throughput. The work presented in [18] focused on the detection of intrusions in underwater WSNs, employing convolutional LSTM networks with NADAM optimization to achieve superior detection precision and precision. Meanwhile, the research work presented in [19] proposed a trust model utilizing deep reinforcement learning and random forest algorithms to mitigate malicious nodes in underwater sensor networks. The research in [20] integrated Harris Hawks optimization with gradient boost to enhance threat detection in hybrid WSNs, demonstrating exceptional precision on data sets such as NSL-KDD and WSN-DS. Furthermore, the research in [21] presented a federated machine learning framework integrated with blockchain to detect DDoS attacks in IoT networks, incorporating dynamic mining selection for high accuracy and reduced latency.

In terms of hybrid encryption models, studies such as those of [3,12] combined AES and ECC to leverage the security benefits of AES with the efficiency of ECC. Although these models demonstrated superior energy efficiency, they were limited by their static nature and inability to adapt based on real-time feedback. Our proposed model aims to bridge this gap by integrating reinforcement learning for adaptive scaling of the encryption level, which dynamically adjusts the encryption strength according to network conditions and threat levels. This approach is inspired by the work of [2], who successfully demonstrated that reinforcement learning could optimize routing protocols in WSNs.

In addition, some research has explored the combination of reinforcement learning with blockchain to improve security in distributed WSNs. In particular, ref. [14] introduced a blockchain-enhanced reinforcement learning framework that provides decentralized security while dynamically adjusting encryption parameters. Although blockchain integration is beneficial for distributed networks, its computational requirements may be excessive for energy-constrained WSN nodes [13].

### 2.1. Reinforcement Learning Models for Attack Mitigation

Several works have specifically focused on Q-learning in the context of adaptive security for WSNs. The researchers of [13] proposed a Q-learning-based model to adjust encryption levels based on network activity and resource availability. Their model showed improved adaptability and energy efficiency compared to fixed encryption schemes, although its security effectiveness against evolving threats remained a concern. Subsequently, ref. [1] enhanced this model by introducing reward functions that prioritize security in high-risk scenarios. However, their approach lacked a comprehensive evaluation of latency and packet delivery, which are critical metrics for time-sensitive WSN applications. These vulnerabilities compromise the security, reliability, and energy efficiency of data communication in these systems [17,19,22,23,24]. Current security solutions often face limitations in scalability, adaptability to dynamic environments, and computational efficiency [20,21,25,26,27].

Suhag and Aarti [22] highlighted the critical challenges in securing WSNs, proposing asymmetric cryptographic encryption as an effective strategy to protect data in transit from impersonation and compromise attacks. Devi and Kumar [23] presented a secure data framework based on key reconciliation to improve the reliability of data exchange in WSNs, addressing key management and economic incentives. Similarly, Yesodha et al. [24] combined trust modeling with ant colony optimization in an ECC-based secure routing protocol to improve energy efficiency and trust analysis in cluster head selection. In parallel, Jagwani and Poornima [25] explored machine learning techniques to detect WSN-specific attacks such as DOS, R2L, and U2R, utilizing the SMOTE technique for data balancing and comparing algorithms using metrics such as MCC and F1 scores.

Further advances in smart environments include the Aziz and Mirzaliev decision tree [26] and the gray wolf optimization model for robust intrusion detection in IoT scenarios. Kumar and Kumar [27] developed a hybrid encryption and attack detection framework for smart cities enabled by the IoT, integrating deep learning and blockchain to optimize encryption time and enhance attack detection. Altaweel et al. [8] conducted a comprehensive review of security threats in opportunistic mobile networks, proposing strategies to counteract black-hole, wormhole, and DDoS attacks. Singh et al. [28] introduced a hybrid machine learning model combining SVM and RF algorithms for DDoS detection in SDN networks, while Ramalakshmi and Kavitha [29] devised a distributed multi-controller approach to mitigate DDoS attacks in fog-based IoT systems. In particular, Han et al. [30] proposed a trust-aware clustering algorithm to address trust-based attacks, and Saleh et al. [31] investigated the integration of blockchain and machine learning to address security issues in medical applications of IoT. These studies collectively underscore ongoing innovations aimed at fortifying WSNs and IoT networks against emerging cybersecurity threats.

### 2.2. Adaptive Encryption Models for Attack Mitigation

WSNs have gained significant attention due to their ability to collect and transmit data in various applications, from environmental monitoring to military operations [1]. Given the limited energy resources of the WSN nodes, ensuring security without compromising efficiency is a critical challenge. Traditional encryption techniques, such as AES and RSA, while effective in securing communication, often consume substantial computational resources, which is a constraint for WSN environments [5]. To address these limitations, numerous studies have focused on lightweight and adaptive encryption schemes, particularly integrating machine learning and, more recently, reinforcement learning, to optimize encryption protocols based on network conditions [3].

Several researchers have explored lightweight cryptographic algorithms as potential solutions for resource-constrained environments such as WSNs. Studies such as those by [4,6] proposed variants of lightweight block ciphers designed to reduce encryption overhead while maintaining acceptable levels of security. Similarly, elliptic curve cryptography (ECC) has been frequently studied due to its smaller key size requirements, which offer both energy efficiency and security [11]. However, static approaches to lightweight encryption often lack the flexibility to adapt to varying security requirements in real-time.

To enable adaptive security mechanisms, some researchers have integrated threshold-based encryption models. The work of [12] introduced dynamic threshold encryption, which adjusts the encryption level based on pre-determined threshold values of network parameters. Although effective, such models still rely on fixed thresholds, which may not account for rapidly changing network conditions, especially in dynamic WSNs. In contrast, reinforcement learning, particularly Q-learning, has shown promise in addressing these limitations by allowing real-time decision-making [2].

A growing body of literature has applied machine learning techniques to improve WSN security protocols. In their seminal work, ref. [14] explored the integration of supervised learning for anomaly detection, enabling the identification of potential threats with minimal energy consumption. Recent advances, as seen in [15], have extended this approach to unsupervised learning, allowing the detection of novel threats without prior labeled data. However, machine learning models generally require substantial computational resources, which can limit their feasibility in resource-constrained WSN environments. Reinforcement learning, specifically Q-learning, offers a promising solution by dynamically adapting encryption levels in real time while considering energy constraints, as explored in [5].

### 2.3. Issues with Existing Models

The research analyzed emphasizes the limitations of using static encryption methods in WSNs and points to the promise of reinforcement learning in achieving adaptive encryption. A summary of the existing models with an overview comparison of the previous works indicating the methods, advantages, challenges, limitations, and experimental results is presented in Table 1. This research extends these foundational studies by introducing a Q-learning-driven encryption level adjustment strategy, which dynamically modulates encryption strength based on real-time network condition feedback. This novel approach aims to provide an efficient balance between energy consumption, latency, and security, which has been insufficiently addressed in previous works.

As WSNs and IoT systems are increasingly integrated into critical domains such as smart cities, healthcare, underwater networks, and industrial automation, these challenges pose a significant threat to the integrity of the data and the functionality of systems. To address these issues, there is an urgent need for advanced security frameworks that leverage cutting-edge technologies such as blockchain, machine learning, and optimization techniques to improve resilience and adaptability [8,28,29,30,31]. Developing hybrid approaches that integrate cryptographic mechanisms, trust models, and intelligent threat detection algorithms will be essential to effectively secure WSN and IoT ecosystems.

### 2.4. Research Gaps Identified

Several studies have explored machine learning applications in WSNs, particularly for anomaly detection and intrusion prevention. For example, supervised learning techniques have been used to detect unauthorized access or abnormal patterns, effectively identifying potential security threats. However, these approaches typically require labeled data for training, which may not always be available or feasible in WSN environments. On the other hand, unsupervised learning models, while useful for anomaly detection without labeled data, often lack the adaptability needed to adjust encryption in real time. Reinforcement learning addresses these limitations by enabling an autonomous learning process based on trial and error, where the model learns to select optimal encryption levels by maximizing rewards associated with energy savings and threat mitigation. This dynamic adaptability to real-time network conditions represents a significant advancement over static and semistatic encryption schemes.

Despite progress in lightweight and adaptive encryption techniques, current methodologies often lack comprehensive solutions to balance energy efficiency with robust security measures in dynamic WSN environments. Most existing encryption schemes focus on either energy optimization or security enhancement, but seldom address both requirements concurrently. Furthermore, current adaptive encryption methods, particularly threshold-based approaches, lack the flexibility to respond to rapidly changing network conditions and fail to optimize encryption levels based on real-time feedback. Consequently, there exists a research gap in developing a truly adaptive and energy-efficient encryption protocol capable of scaling encryption levels based on real-time network and threat conditions.

The proposed research aims to address this gap by introducing a reinforcement learning-based adaptive encryption model for WSNs. This model utilizes Q-learning to dynamically adjust encryption levels in response to real-time feedback on energy availability, data sensitivity, and threat level. Through this approach, the proposed method can optimize encryption based on current conditions, thereby enhancing energy efficiency while ensuring robust security. Furthermore, the model incorporates a reward system that balances the trade-offs between energy consumption, latency, and security, ensuring that the encryption scheme is both effective and efficient.

## 3. Reinforcement Q-Learning Framework for Adaptive Encryption

This research introduces a reinforcement learning framework designed to dynamically modify encryption levels in WSNs, with the aim of enhancing energy efficiency without compromising security. The framework adapts encryption levels based on real-time feedback from the network environment, allowing each sensor node to balance between energy consumption and data protection based on immediate needs. The methodology integrates modern approaches such as double Q-learning and dynamic parameter adjustment to enhance scalability, security, and energy efficiency in resource-constrained environments.

### 3.1. Methodology

**Figure 1:**
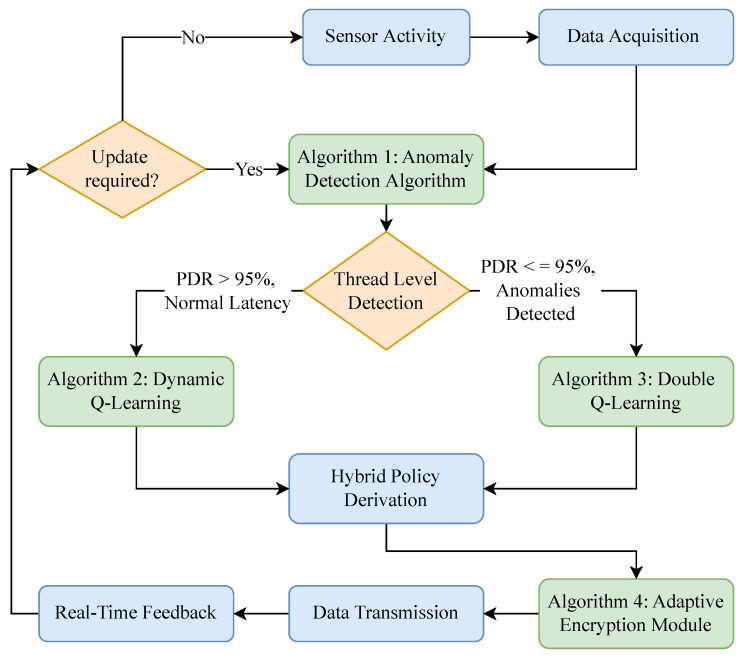
Process flow of proposed enhanced adaptive encryption.

The proposed reinforcement learning framework addresses the challenges of secure and energy-efficient data transmission in WSNs. The learning agent operates in a dynamic environment where states represent network conditions and actions involve adaptive encryption decisions. The framework adopts double Q-learning to mitigate the overestimation bias of traditional Q-learning, as shown in Algorithm 3. This enhancement ensures robustness in selecting optimal actions under varying network conditions. Furthermore, the dynamic exploration rate (ϵ) ensures effective exploration during initial episodes and shifts toward exploitation in later stages. Figure 1 illustrates the process flow of an adaptive encryption methodology for WSNs. It integrates anomaly detection (Algorithm 4), dynamic Q-learning (Algorithm 1), double Q-learning (Algorithm 2), and hybrid policy derivation (Algorithm 3). The diagram represents the flow of data and decision-making across various components, highlighting how the system adapts encryption levels based on real-time network conditions.

The process begins with sensor activity, where environmental and network data are collected and processed through the data acquisition module. This data includes key metrics such as PDR, latency, packet drop rate, and traffic volume. These metrics are used to detect anomalies and classify the state of the network into low or high risk levels. The thread-level detection component acts as the decision node, relying on thresholds to assess the PDR and detect anomalies. If the PDR exceeds 95% and the latency remains normal, the system categorizes the situation as a low-threat condition. In contrast, if the PDR drops below 95% or anomalies are detected, the network is classified as operating under high threat.

The anomaly detection algorithm (Algorithm 4) leverages a feedforward neural network (FNN) to compute an anomaly score based on input metrics. This score quantifies deviations from normal network patterns, allowing the system to make informed decisions about threat levels. When the anomaly score and the metrics indicate low-threat conditions, dynamic Q-learning (Algorithm 1) is activated to optimize energy efficiency. This algorithm dynamically scales encryption levels to balance security needs with resource conservation. For high-threat conditions, double Q-learning (Algorithm 1) is triggered to improve security. This algorithm uses two Q-tables to mitigate overestimation bias and selects robust encryption levels to counter potential cyberattacks.

The hybrid policy derivation module (Algorithm 3) integrates the outputs of dynamic Q-learning and double Q-learning to form a unified encryption policy. For low-threat conditions, the policy prioritizes energy-efficient actions derived from Algorithm 1. For high-threat conditions, it prioritizes secure actions from Algorithm 2. The resulting hybrid policy ensures that the system adapts dynamically to varying threat levels, balancing energy efficiency and security robustness. Once the hybrid policy is established, the adaptive encryption module applies the selected encryption levels to the data. This ensures that data are securely encrypted while minimizing unnecessary resource usage. The encrypted data are then transmitted across the network through the data transmission module. Real-time feedback from this transmission process is looped back into the system to update the Q-tables and refine the anomaly detection model, enabling continuous learning and improvement.

### 3.2. System Model and Assumptions

The wireless sensor network is represented as a set of nodes $N=\{n_{1},n_{2},\cdots ,n_{m}\}$, each capable of sensing, processing, and transmitting data. Each node $n_{i}\in N$ has a limited energy supply $E_{i}$, and its primary objective is to conserve this energy while ensuring secure data transmission. A summary of the abbreviations and acronyms used in this work is presented in Table 2. The level of security risk of the environment and the energy state of each node evolve over time, which we represent as an MDP [16] defined by a tuple 〈S,A,P,R〉, where the following definitions hold:

- *S* is the state space that captures the energy levels of the nodes and the levels of threat of the network.
- *A* is the action space that corresponds to possible encryption levels.
- *P* is the state transition probability, denoted as $P(s^{\prime }|s,a)$.
- *R* is the reward function that balances energy efficiency and security.

**State Space:** The state s∈S represents the current conditions of the node, encapsulating the factors that influence encryption decisions. We define *s* as a vector presented as $s=\left[E_{i},\;T_{net}\right]$. Here, $E_{i}$ is the current energy level of node $n_{i}$, and $T_{net}$ is a measure of the current threat level in the network, which is evaluated based on the frequency and intensity of the detected security events. The state space *S*, therefore, consists of discrete values for $E_{i}$ and $T_{net}$, allowing the agent to estimate the impact of its actions on both energy and security.

**Action Space:** The action space *A* represents the set of available encryption levels for the sensor nodes. Each action a∈A corresponds to a choice of encryption strength, ranging from lightweight to strong encryption. The action space can be mathematically defined as $A=\{a_{1},a_{2},\cdots ,a_{k}\}$, where each $a_{j}$ denotes a specific encryption level. Higher values of $a_{j}$ indicate stronger encryption, which provides increased security but consumes more energy. At each time step *t*, the nodes select an action $a_{t}$ based on the current state $s_{t}$. This selection process is designed to optimize energy usage while dynamically responding to perceived threat levels in the network.

**Reward Function:** The reward function R(s,a) is a critical component of the learning process, which guides the sensor nodes to achieve an optimal balance between security and energy consumption. This is achieved by dynamically adjusting the encryption level based on real-time network conditions, ensuring robust security while minimizing computational overhead. This reward function for the proposed model is defined as $R\left(s,a\right)=-\alpha \cdot E_{\mathrm{enc}}\left(a\right)+\beta \cdot S_{\mathrm{eff}}\left(a,T_{net}\right)$, where $E_{\mathrm{enc}}\left(a\right)$ represents the energy consumption associated with the encryption level *a*, and $S_{\mathrm{eff}}\left(a,T_{net}\right)$ denotes the security effectiveness of the selected encryption level *a* given the current threat level $T_{net}$. The coefficients α and β are weighting parameters that control the trade-off between energy efficiency and security robustness.

The function $E_{\mathrm{enc}}\left(a\right)$ is generally a monotonically increasing function, reflecting the fact that higher encryption levels consume more energy. On the other hand, $S_{\mathrm{eff}}\left(a,T_{net}\right)$ increases with both the encryption level and the threat level, ensuring that stronger encryption measures are applied when security risks are elevated. This reward structure incentivizes nodes to dynamically adapt their encryption levels, prioritizing security in high-threat scenarios while conserving energy during normal operations. The reward function in the proposed model dynamically balances security and energy efficiency by adjusting encryption levels based on real-time network conditions. When the threat level is high, the reward prioritizes stronger encryption to enhance security, even at the cost of higher energy consumption. In low-threat scenarios, it penalizes excessive encryption to conserve energy while maintaining adequate security. Through Q-learning updates, the model continuously optimizes encryption decisions, ensuring maximum cyberattack mitigation (94%), energy efficiency (30.5% reduction), and a high packet delivery ratio (92.5%). This adaptive approach optimally manages security and resource constraints in WSNs.

### 3.3. Deep Learning-Based Anomaly Detection

Anomaly detection plays a crucial role in identifying cyber threats in WSNs. In this research, a deep learning-based approach is used to detect anomalies in network traffic, ensuring robust differentiation between low- and high-threat scenarios. This method uses neural networks to analyze real-time data and identify deviations from normal patterns. PDR serves as a critical indicator of network performance, with anomaly detection focusing on variations in PDR and other key metrics. The proposed method uses a feedforward neural network (FNN) trained on historical network data to classify traffic as normal or anomalous. The network analyzes characteristics such as PDR, latency, packet drop rate, and traffic volume to compute an anomaly score. Based on this score, the system determines whether the current state represents a high or low threat.

Algorithm 4 outlines the steps for anomaly detection using a deep learning-based approach. The algorithm dynamically evaluates the network conditions to compute an anomaly score and classifies the threat level accordingly.

**Process to Detect Anomalies:** First, real-time network data are collected, including metrics such as PDR, latency, packet drop rate, and traffic volume. These metrics are pre-processed and normalized before being fed into the deep learning model. The feedforward neural network model M computes an anomaly score *A* based on input data. This score quantifies the deviation of current network conditions from historical patterns.

If the anomaly score *A* is less than or equal to a predefined lower threshold $\tau_{\mathrm{low}}$, the system classifies the situation as a low threat and triggers energy-efficient actions using Algorithm 4. In contrast, if *A* exceeds a higher threshold $\tau_{\mathrm{high}}$, the situation is classified as high threat, and robust encryption scaling is initiated using Algorithm 1. For anomaly scores between $\tau_{\mathrm{low}}$ and $\tau_{\mathrm{high}}$, the system considers it a moderate threat and balances actions between energy efficiency and security.

**PDR Analysis:** PDR serves as a primary metric in the anomaly detection process, allowing the system to monitor variations in network performance and identify potential threats. A PDR greater than 95% typically indicates normal operating conditions with no significant threats present. When the PDR falls between 90% and 95%, it suggests the presence of moderate anomalies that warrant attention. A PDR below 90% signals a high threat level, which may result from attacks such as DDoS or packet injection. By integrating deep learning techniques with PDR analysis, the proposed approach achieves accurate and dynamic anomaly detection, enabling the system to adapt effectively to real-time network environments.

### 3.4. Q-Learning Algorithm

The Q-learning algorithm is a model-free value-based reinforcement learning method that enables agents (sensor nodes) to learn optimal policies for decision-making in a dynamic environment. In this context, the agent adapts encryption levels based on real-time observations of the network, such as node energy levels and security threat levels. This section describes the mathematical foundations behind the Q-learning algorithm, detailing how Q-values are updated and optimized for secure and energy-efficient operation.

**Q-Value Definition:** In Q-learning, the quality of a particular action *a* taken in a state *s* is represented by the Q-value Q(s,a). This Q-value estimates the expected cumulative reward from the execution of the action *a* in state *s*, considering the future rewards that can be obtained by following an optimal policy from that point on. Mathematically, the Q-value is expressed as Equation (1).

(1) $$Q\left(s,a\right)=E\left[\sum_{k=0}^{\infty }\gamma^{k}r_{t+k+1}\;|\;s_{t}=s,\;a_{t}=a\right]$$

where:

- $r_{t+k+1}$ is the reward received k+1 steps after taking action *a* in state *s* at time *t*.
- γ∈[0,1] is the discount factor, which determines the importance of future rewards relative to immediate rewards.

**Bellman Equation and Q-Value Update:** Q-learning updates the Q-values using the Bellman equation, which recursively defines the value of taking an action in a given state in terms of the immediate reward plus the discounted value of the best action in the subsequent state. The Bellman equation for Q-learning is given in Equation (2).

(2) $$Q\left(s,a\right)=r+\gamma \max_{a^{\prime }}Q\left(s^{\prime },a^{\prime }\right)$$

where:

- *r* is the immediate reward for taking action *a* in state *s*.
- $s^{\prime }$ is the next state resulting from action *a*.
- $\max_{a^{\prime }}Q\left(s^{\prime },a^{\prime }\right)$ is the maximum Q-value achievable from state $s^{\prime }$.

In practice, the Q-value is updated incrementally as new state–action–reward transitions are observed. The update rule for Q-learning follows Equation (3).

(3) $$Q\left(s_{t},a_{t}\right)\leftarrow Q\left(s_{t},a_{t}\right)+\alpha \left[r_{t}+\gamma \max_{a^{\prime }}Q\left(s_{t+1},a^{\prime }\right)-Q\left(s_{t},a_{t}\right)\right]$$

where

- α∈(0,1] is the learning rate, which determines the extent to which newly acquired information overrides the old information.
- $r_{t}$ is the reward received after taking action $a_{t}$ in state $s_{t}$.
- γ is the discount factor, as defined earlier.

This update rule ensures that $Q(s_{t},a_{t})$ converges to the expected cumulative reward over time, provided that all state–action pairs are visited an infinite number of times and α gradually decreases.

**Convergence of Q-Learning:** The Q-learning algorithm is proven to converge to the optimal Q-values $Q^{*}\left(s,a\right)$ under certain conditions. Formally, if each state–action pair (s,a) is visited infinitely often and the learning rate α satisfies, as given in Equation (4),

(4) $$\sum_{t=1}^{\infty }\alpha_{t}=\infty \;\mathrm{and}\;\sum_{t=1}^{\infty }\alpha_{t}^{2}<\infty$$

then $Q\left(s,a\right)\to Q^{*}\left(s,a\right)$ as t→∞. Here, $Q^{*}\left(s,a\right)$ represents the optimal Q-value, which satisfies the Bellman optimality Equation (5):

(5) $$Q^{*}\left(s,a\right)=E\left[r+\gamma \max_{a^{\prime }}Q^{*}\left(s^{\prime },a^{\prime }\right)\right]$$

**Optimal Policy Extraction:** Once the Q-values converge to $Q^{*}\left(s,a\right)$, the agent can derive an optimal policy $\pi^{*}$ by selecting the action with the highest Q-value in each state. The optimal policy is given as Equation (6).

(6) $$\pi^{*}\left(s\right)=\arg\max_{a}Q^{*}\left(s,a\right)$$

For each state s∈S, the agent chooses the action that maximizes the expected cumulative reward. The policy $\pi^{*}$ represents the optimal strategy for scaling the adaptive encryption level in the wireless sensor network, balancing energy consumption and security based on real-time conditions.

**Exploration vs. Exploitation: ϵ-Greedy Policy:** During the learning phase, the agent employs an ϵ-greedy policy to balance exploration (trying new actions) and exploitation (choosing the best known action). The ϵ-greedy policy is defined in Equation (7).

(7) $$a_{t}=\left\{\begin{array}{cc} \arg\max_{a}Q\left(s_{t},a\right), & \mathrm{with}\;\mathrm{probability}\;1-\epsilon \\ \mathrm{random}\;\mathrm{action}\;a\in A, & \mathrm{with}\;\mathrm{probability}\;\epsilon \end{array}\right.$$

where ϵ∈[0,1] is a parameter that controls the degree of exploration. A higher value of ϵ encourages exploration of new actions, while a lower ϵ favors exploitation of the current knowledge.

**Temporal Difference (TD) Error:** The temporal difference (TD) error, denoted $\delta_{t}$, measures the difference between the current Q-value and the updated Q-value based on the observed reward and the estimated future reward. The TD error is computed using Equation (8).

(8) $$\delta_{t}=r_{t}+\gamma \max_{a^{\prime }}Q\left(s_{t+1},a^{\prime }\right)-Q\left(s_{t},a_{t}\right)$$

This error term $\delta_{t}$ is used to update the Q-value, allowing the agent to correct its estimates over time based on newly observed rewards.

**Final Q-Learning Update Rule with TD Error:** Combining the update rule and TD error, the Q-learning update equation can be rewritten as given in Equation (9).

(9) $$Q\left(s_{t},a_{t}\right)\leftarrow Q\left(s_{t},a_{t}\right)+\alpha \delta_{t}$$

where $\delta_{t}$ is the temporal difference error calculated as above. This form emphasizes that the Q-value is incrementally updated by a factor of the TD error, weighed by the learning rate α. The proposed Q-learning algorithm is enhanced with dynamic exploration rates as given in Equation (10).

(10) $$\epsilon_{t}=\epsilon_{\min}+\left(\epsilon_{\max}-\epsilon_{\min}\right)e^{-\lambda \cdot t}$$

where λ controls the rate of decay, ensuring the agent transitions smoothly from exploration to exploitation over time. Algorithm 2 provides a detailed description of the integration of double Q-learning with this dynamic exploration mechanism.

**Pseudocode for Q-Learning with TD Update:** The updated algorithms, incorporating double Q-learning and dynamic parameter adjustment, are detailed in Algorithms 1 and 2.

Through repeated training, each node learns to choose optimal encryption levels based on its current energy state and the perceived threat level in the network. This reinforcement learning-based approach enables dynamic, energy-efficient adaptation to real-time security needs, effectively balancing the trade-offs between energy consumption and data protection.

**Interpretation of Q-Values in the Context of Adaptive Encryption:** In the proposed reinforcement learning framework, the Q-value Q(s,a) represents the balance between energy efficiency and security effectiveness for a given pair of actions of state (s,a). As nodes update Q-values over time, they learn to associate specific encryption levels (actions) with particular network states, achieving optimal encryption selection based on the node’s energy level and network threat conditions.

This Q-learning algorithm allows nodes to adaptively adjust their encryption levels in real time, ensuring that high security is applied during elevated threat levels while conserving energy when the environment is relatively secure. This dynamic adjustment ultimately improves the longevity and resilience of the wireless sensor network.

**Policy and Action Selection:** We employ an ϵ-greedy policy for action selection, where the node explores new actions with a probability ϵ and exploits its knowledge by choosing the action with the highest Q-value with probability 1−ϵ. This approach ensures a balance between exploration (trying new encryption levels) and exploitation (choosing known energy-efficient levels), thus improving the convergence of the Q-learning process. Policies are derived from the Q-values to ensure optimal action selection using Equation (11).

(11) $$\pi \left(s\right)=\arg\max_{a}Q\left(s,a\right)$$

For double Q-learning, actions are determined using both Q-tables to mitigate bias using Equation (12).

(12) $$\pi \left(s\right)=\arg\max_{a}\left[Q_{1}\left(s,a\right)+Q_{2}\left(s,a\right)\right]$$

## 4. Adaptive Cyberattack Mitigation with Hybrid Encryption

The proposed adaptive encryption Algorithm 3 is designed to dynamically mitigate cyberattacks by scaling encryption levels based on real-time network conditions and detected threats. It integrates Q-learning and double Q-learning methodologies to ensure robust policy learning while adapting to evolving network scenarios.

The algorithm operates using a well-defined state and action space. The state (*s*) represents critical network metrics such as detected threat levels, residual energy of the nodes, PDR, and latency. These metrics provide the agent with a comprehensive understanding of the current condition of the network. The action (*a*) space includes three encryption levels: no encryption, medium encryption, and full encryption. No encryption minimizes energy usage but provides the least security, medium encryption balances energy efficiency and security, and full encryption maximizes security at the cost of higher energy consumption. This action space allows the algorithm to adaptively select the most appropriate encryption level depending on the situation.

The reward function in this algorithm is carefully designed to balance security and efficiency. During high-threat scenarios, such as DDoS or data injection attacks, the algorithm prioritizes security by assigning higher rewards to actions that enhance encryption levels. Moreover, weaker encryption actions are more heavily penalized to discourage inadequate responses. Under normal network conditions, the reward function shifts focus to optimizing energy efficiency and reducing latency, thus preserving resources without compromising performance.

Dynamic exploration is a key feature of the algorithm, enabling it to balance the trade-off between exploration and exploitation. At the beginning of the learning process, a high exploration rate (ϵ) allows the agent to investigate various strategies to identify optimal actions. As learning progresses, the exploration rate decays exponentially according to the following Equation (13):

(13) $$\epsilon_{t}=\epsilon_{\min}+\left(\epsilon_{\max}-\epsilon_{\min}\right)e^{-\lambda \cdot t}$$

where $\epsilon_{\min}$ and $\epsilon_{\max}$ are the minimum and maximum exploration rates and λ controls the decay rate. This decay ensures that the agent transitions smoothly from exploring new strategies to exploiting learned policies, allowing it to adapt effectively to changing network conditions.

The algorithm’s threat handling mechanism dynamically adjusts encryption levels based on the detected threat level. In high-threat scenarios, the algorithm favors resource-intensive encryption actions to maximize security. Conversely, during low-threat periods, it focuses on conserving resources by selecting less demanding encryption levels. This adaptability ensures robustness and efficiency under diverse operating conditions. Double Q-learning plays a central role in the algorithm, addressing the overestimation bias inherent in traditional Q-learning. The algorithm maintains two Q-tables, $Q_{1}$ and $Q_{2}$, which work together to ensure unbiased policy updates. When updating the Q-tables, the following Equations (14) and (15) are used:

(14) $$Q_{1}\left(s,a\right)\leftarrow Q_{1}\left(s,a\right)+\alpha \left[r+\gamma Q_{2}\left(s^{\prime },\arg\max_{a^{\prime }}Q_{1}\left(s^{\prime },a^{\prime }\right)\right)-Q_{1}\left(s,a\right)\right]$$

(15) $$Q_{2}\left(s,a\right)\leftarrow Q_{2}\left(s,a\right)+\alpha \left[r+\gamma Q_{1}\left(s^{\prime },\arg\max_{a^{\prime }}Q_{2}\left(s^{\prime },a^{\prime }\right)\right)-Q_{2}\left(s,a\right)\right]$$

Here, α is the learning rate, γ is the discount factor, *r* is the observed reward, and $s^{\prime }$ is the next state. These equations ensure that the Q-values are updated accurately by leveraging both Q-tables alternately, thereby mitigating overestimation bias. The final policy π(s) is derived by combining the values of both Q tables to determine the optimal action for a given state. This is expressed as Equation (16).

(16) $$\pi \left(s\right)=\arg\max_{a}\left[Q_{1}\left(s,a\right)+Q_{2}\left(s,a\right)\right]$$

This approach enhances the reliability of the learned policy, making it more robust in selecting optimal actions under varying conditions. The proposed algorithm is capable of dynamically responding to threats by scaling encryption levels to ensure robust mitigation of cyberattacks. The integration of double Q-learning guarantees accurate and unbiased policy learning, as shown in Equations (14) and (15). Furthermore, the use of dynamic exploration, as described in Equation (13), allows the algorithm to adapt efficiently to the changing demands of the network environment. Together, these features make the algorithm an effective solution for achieving a balance between security, energy efficiency, and latency in minimizing cyberattacks.

## 5. Performance Evaluation

To evaluate the performance of the proposed reinforcement learning-based adaptive encryption mechanism, we conducted a series of experiments in a simulated WSN environment using MATLAB Simulink R2022b. This section provides a detailed description of the metrics used for evaluation, the dataset used, and the results obtained from the experiments.

**Dataset:** The experiments were carried out using a synthetic dataset designed to simulate real-world scenarios in wireless sensor networks. The dataset included parameters such as the number of sensor nodes, threat levels, energy levels, and simulation duration, as shown in Table 3.

**Figure 2:**
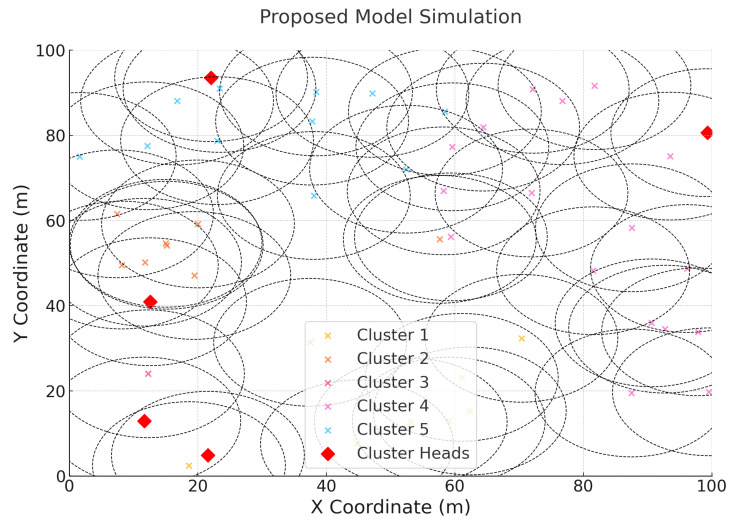
Simulation of WSNs.

The network included 50 sensor nodes, each initialized with unique energy levels drawn from a uniform distribution ranging between 10J and 100J. Threat levels were simulated as random attack intervals classified as low, medium, and high, which influenced the security decisions of the nodes. The simulation was carried out over a duration of 300 s, during which the nodes continuously transmitted data while adapting their encryption levels based on the policies learned through reinforcement learning. The simulation graph is presented in Figure 2. As per the simulation setup used, transmitting 100 packets of 1024 bytes each from 50 sensor nodes over a ZigBee-based WSN requires approximately 170 s. To evaluate each packet against different attacks using the proposed model, a computational overhead of 20 ms per packet along with a latency of 2 ms per packet adds an additional 100 s. Thus, the total simulation time is set to 300 s (270 s required on average). Any variation in simulation time is directly proportional to the number of packets transmitted.

### 5.1. Metrics for Evaluation

The effectiveness of the adaptive encryption algorithm was assessed using several key performance metrics. **Energy consumption** was measured to evaluate the impact of encryption decisions on the lifetime of sensor nodes. The total energy consumed by a sensor node during data transmission is given as Equation (17).

(17) $$E=\sum_{i=1}^{N}E_{i}$$

where $E_{i}$ represents the energy consumed for each transmission *i*, and *N* is the total number of transmissions during the experiment. **Packet Delivery Ratio (PDR)** was used as a measure of communication reliability, defined in Equation (18).

(18) $$\mathrm{PDR}=\frac{\mathrm{Number}\;\mathrm{of}\;\mathrm{Packets}\;\mathrm{Delivered}}{\mathrm{Total}\;\mathrm{Number}\;\mathrm{of}\;\mathrm{Packets}\;\mathrm{Sent}}\times 100$$

Higher PDR values indicate greater reliability, which is critical to ensuring the effectiveness of adaptive encryption. **Latency**, another key metric, was measured as the time it took a data packet to travel from the source to the destination, as given in Equation (19).

(19) $$L=T_{arrival}-T_{departure}$$

where $T_{arrival}$ and $T_{departure}$ denote the arrival and departure timestamps of the packet, respectively. Finally, the metric of Security Effectiveness quantified the network’s resilience to threats, defined as Equation (20).

(20) $$\mathrm{Security}\;\mathrm{Effectiveness}=\frac{\mathrm{Number}\;\mathrm{of}\;\mathrm{Threats}\;\mathrm{Detected}}{\mathrm{Total}\;\mathrm{Number}\;\mathrm{of}\;\mathrm{Threat}\;\mathrm{Attempts}}\times 100$$

This metric provides valuable information on how well the proposed encryption mechanism can mitigate security threats in real time.

### 5.2. Comparison Analysis and Discussion

To comprehensively evaluate the performance of the proposed reinforcement learning-based adaptive encryption model, we conducted a comparative analysis with six established models in wireless sensor networks. Each model was evaluated based on four key metrics: energy consumption, PDR, latency, and security effectiveness. These metrics provide a holistic view of each model’s ability to balance security, efficiency, and responsiveness under dynamic network conditions. The six baseline models used in the comparison are the following:

- **AES-128 Fixed Encryption:** Standard fixed 128-bit encryption, widely used but non-adaptive.
- **Lightweight Block Cipher (LBC):** Optimized for low-energy environments, with minimal computational overhead.
- **Elliptic Curve Cryptography (ECC):** Public-key encryption with low energy requirements and suitable for constrained devices.
- **Hybrid AES-RSA:** Uses AES for encryption and RSA for key exchange, balancing energy use and security.
- **Dynamic Threshold Encryption (DTE):** An adaptive encryption approach that scales on the basis of estimated threat levels.
- **Blockchain-based Lightweight Encryption (BLE):** Integrates blockchain for enhanced security, suitable for distributed WSN applications.

The proposed model and each baseline model were evaluated for energy consumption, PDR, latency, and security effectiveness. The results are shown in Table 4. Table 4 indicates that the proposed model achieves the lowest energy consumption, the highest packet delivery ratio, the lowest latency, and the highest security effectiveness. This validates the efficiency and adaptability of the reinforcement learning-based approach. Table 4 provides information on the effectiveness of different security mechanisms in Wireless Sensor Networks (WSNs) by evaluating energy consumption in various models. The findings indicate a clear correlation between the level of security applied and the corresponding energy expenditure.

The lowest energy consumption is recorded in the proposed model at 45.2 J, highlighting its efficiency in maintaining security with minimal power usage. In contrast, AES-128 exhibits the highest energy consumption at 72.5 J, demonstrating the computational overhead associated with its encryption complexity. Other models such as DTE (52.8 J) and BLE (60.7 J) achieve moderate energy usage, balancing security and efficiency. The variation in energy consumption can be attributed to the computational burden imposed by different security protocols. AES-128 [33] and H-AES-RSA [34], which employ more resource-intensive cryptographic operations, lead to higher energy usage due to increased processing and memory requirements. On the other hand, the proposed model leverages an adaptive security mechanism that dynamically adjusts encryption complexity based on threat levels, significantly reducing unnecessary computational overhead.

Furthermore, the effectiveness of security measures impacts packet transmission efficiency, further influencing energy consumption. As observed in Table 4, models with lower PDR, such as AES-128 (80.1%), require more retransmissions, leading to higher power consumption. In contrast, the proposed model achieves the highest PDR at 92.5%, reducing the need for packet retransmissions and further conserving energy.

**Figure 3:**
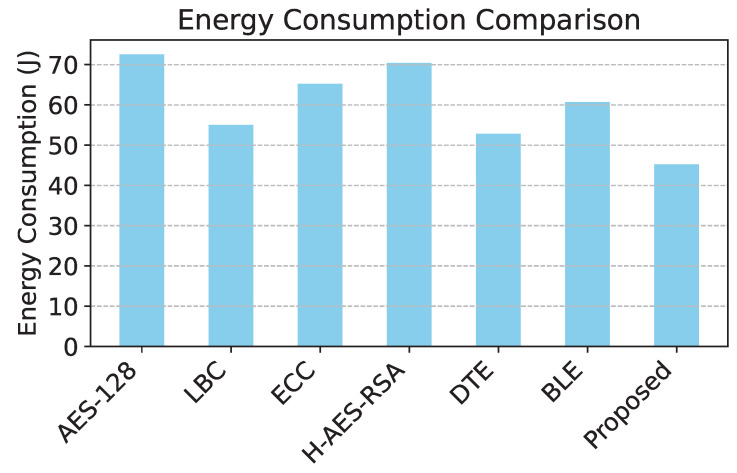
Energy consumption for cyberattacks.

**Figure 4:**
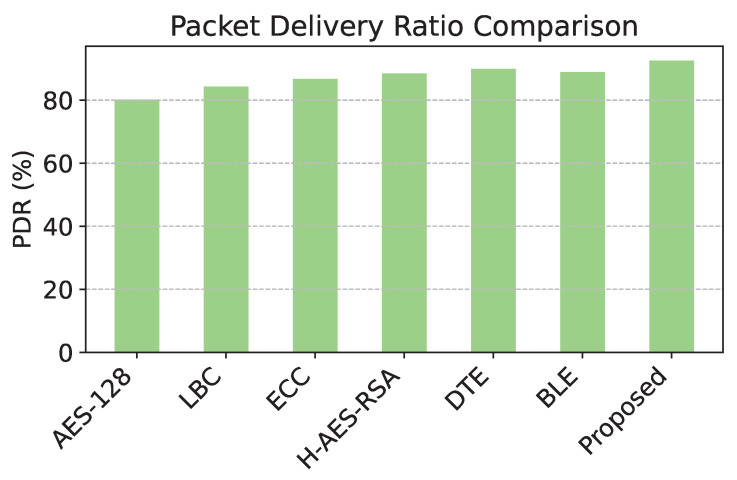
PDR across cyberattacks.

**Figure 5:**
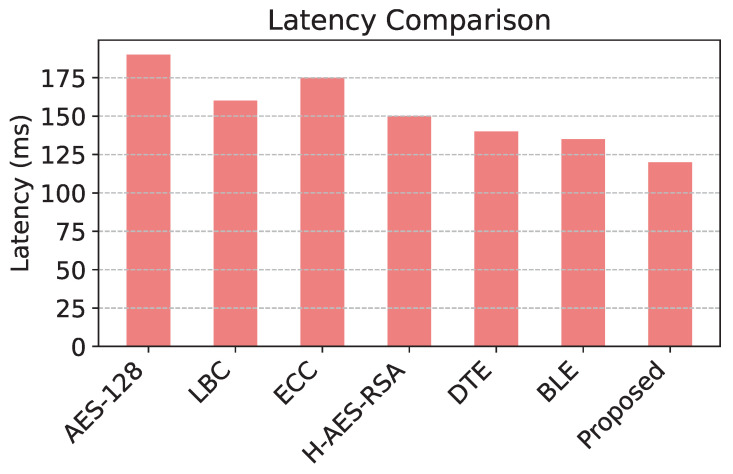
Latency across cyberattacks.

**Figure 6:**
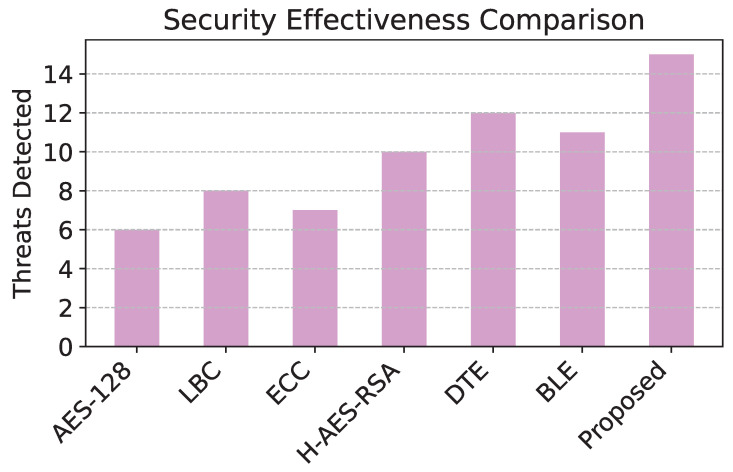
Security effectiveness across cyberattacks.

The comparison of energy consumption in Figure 3 reveals that the proposed model demonstrates significant efficiency, consuming the least energy among all models due to its level of adaptive encryption. Figure 4 shows that the proposed model has the highest packet delivery ratio, demonstrating enhanced reliability and communication efficiency compared to baseline models. The latency comparison in Figure 5 illustrates that the proposed model achieves the lowest latency, making it highly suitable for real-time data transmission in WSNs. The comparison of security effectiveness in Figure 6 indicates that the proposed model detects the highest number of threats, underscoring its superior resilience to network attacks.

The proposed model demonstrates significant improvements in energy efficiency and PDR compared to existing routing protocols. In terms of energy consumption, the proposed model achieves a reduction of 45.2 J, while the highest energy consumption is observed in AES-128 at 72.5 J. This results in an energy reduction of 37.66% compared to AES-128. Even compared to DTE, which has the lowest energy consumption among alternative models (52.8 J), the proposed model still achieves a 14.39% improvement, demonstrating its efficiency in optimizing energy utilization.

Similarly, for PDR, the proposed model attains 92.5%, outperforming AES-128, which records the lowest PDR at 80.1%. This marks an improvement of 15.48% over AES-128. Compared to DTE, which has the highest PDR among alternative models (89.9%), the proposed model still provides an improvement of 2.89%, ensuring enhanced reliability of data transmission. These improvements collectively validate the effectiveness of the proposed approach in reducing energy consumption while maintaining superior network performance.

## 6. Evaluation Against Cyberattack Mitigation

The effectiveness of the proposed reinforcement learning-based adaptive encryption model was assessed through experiments targeting five prevalent cyberattacks in WSNs: DDoS, data injection, black-hole, wormhole, and selective forwarding attacks. Each attack was simulated within a controlled environment to assess the effectiveness of the model in detection and mitigation. The evaluation was performed using three widely used datasets, AWID, IoT-23, and WSN-BFSF, representing various scenarios of the wireless network. The following types of cyberattacks were considered during the evaluation, each representing a significant security challenge in wireless sensor networks:

- **Distributed Denial-of-Service (DDoS) Attack:** Overwhelms the network by flooding it with excessive requests, depleting node resources, and disrupting communication. The goal is to degrade the availability and performance of the network.
- **Data Injection Attack:** Malicious nodes inject falsified or altered data, compromising data integrity and misleading decision-making processes, potentially causing incorrect routing and vulnerabilities.
- **Black-Hole Attack:** A malicious node intercepts and drops all packets, disrupting communication flow, reducing the packet delivery ratio, and compromising network reliability.
- **Wormhole Attack:** Malicious nodes create a tunnel (wormhole) to forward packets to distant parts of the network, bypassing normal routes. This disrupts routing protocols and reroutes traffic through malicious nodes.
- **Selective Forwarding Attack:** A malicious node selectively drops specific packets, targeting particular types of data. This makes the attack harder to detect compared to black-hole attacks.

### 6.1. Experimental Setup for Cyberattack Mitigation

The experiments were carried out using the same simulation setup that was used to evaluate energy consumption, PDR, and latency. The anomaly detection module classified the network states based on key metrics such as PDR, traffic volume, and latency. The model adapted encryption levels dynamically based on the threat classification, balancing energy consumption and security robustness. The experiments used the AWID, IoT-23, and WSN-BFSF datasets to evaluate the generalizability of the model in different network scenarios.

**AWID Dataset (Aegean Wi-Fi Intrusion Dataset) [35]:** This dataset is designed for the detection of intrusion in wireless networks using the IEEE 802.11 [36] (Wi-Fi) protocol. It includes real Wi-Fi traffic labeled as normal or attack types such as injection, impersonation, and DoS. With 154 features related to Wi-Fi traffic, it supports machine learning and deep learning model development for intrusion detection tasks.

**IoT-23 Dataset [37]:** Focused on detecting malicious and benign traffic in IoT networks, this dataset includes 23 captures: 20 for attack scenarios such as botnets and DDoS, and 3 for benign traffic. It features network flow attributes such as timestamps, packet lengths, and IP addresses, providing a rich resource for evaluating intrusion detection systems and traffic classification.

**WSN-BFSF Dataset [38]:** Designed for wireless sensor networks, this dataset targets network layer attacks such as black-hole, flooding, and selective forwarding. With 312,106 labeled instances and 16 features such as energy consumption and packet transmission rates, it is ideal for developing machine learning models to detect cyberattacks and ensure WSN security.

### 6.2. Results and Discussion

**Figure 7:**
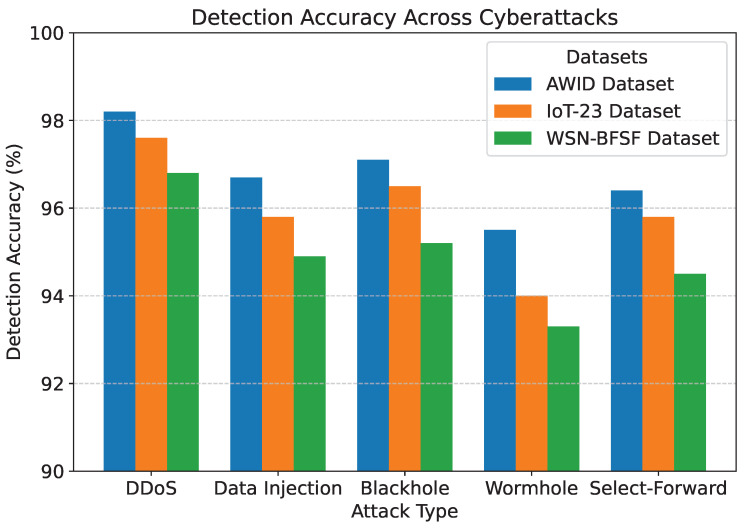
Detection accuracy across cyberattacks.

**Figure 8:**
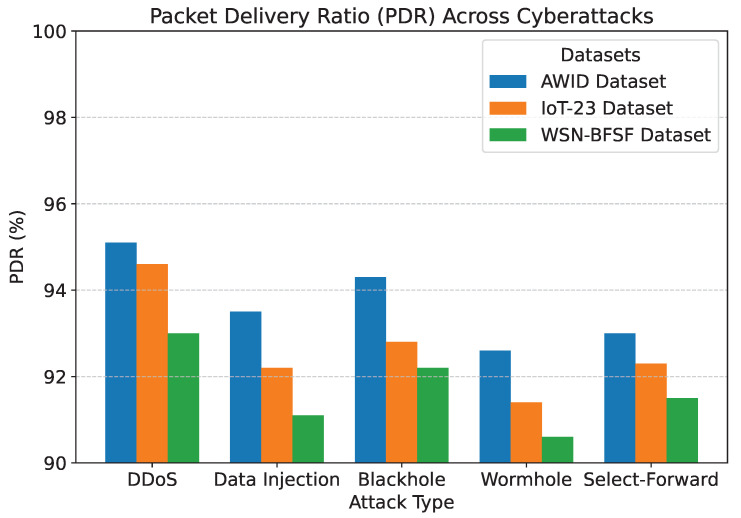
Packet delivery ratio across cyberattacks.

**Figure 9:**
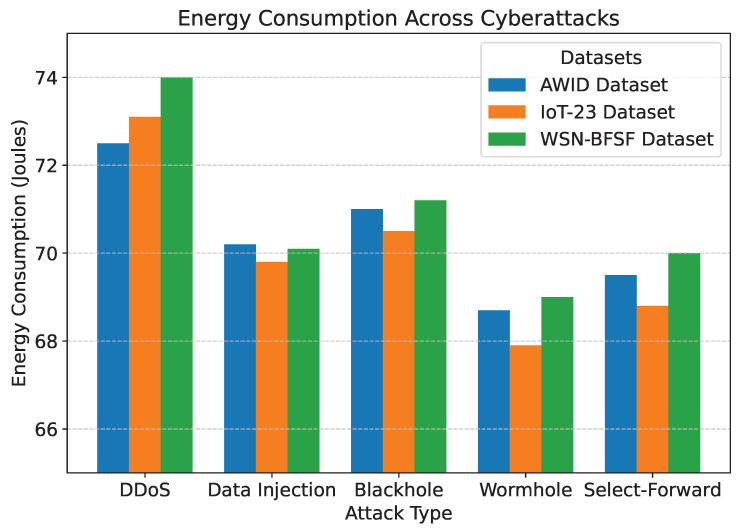
Energy Consumption across cyberattacks.

**Figure 10:**
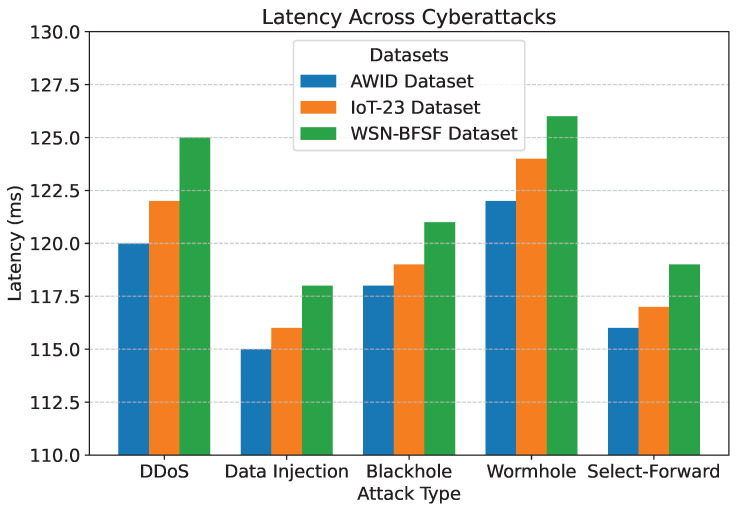
Latency across cyberattacks.

**Figure 11:**
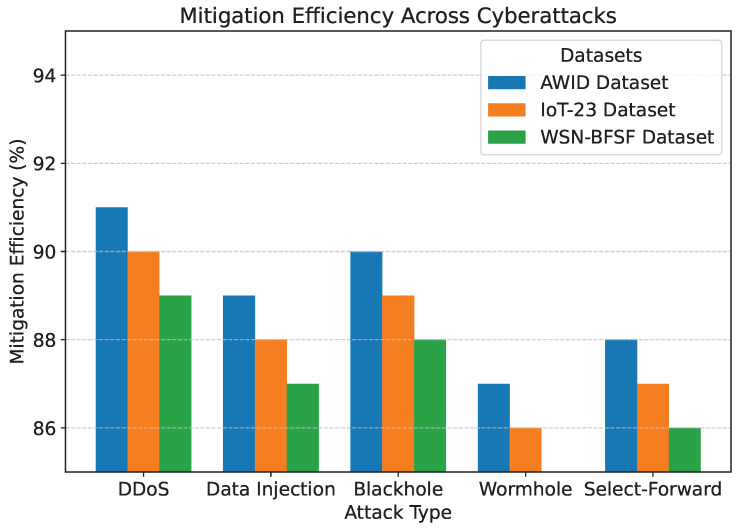
Mitigation efficiency across cyberattacks.

The results of the experiments, summarized in Table 5 and Figure 7, Figure 8, Figure 9, Figure 10 and Figure 11, demonstrate the effectiveness of the proposed model in handling various cyberattacks. Across all datasets, the model consistently achieved high detection accuracy and maintained a strong packet delivery ratio while ensuring energy efficiency and acceptable latency levels. Detailed numercial values are presented in Table 5.

The detection accuracy of the proposed model remained above 94% for all attack scenarios in all datasets, with the highest accuracy observed for the DDoS attack in the AWID dataset at 98.2%. Similarly, the PDR remained above 90%, indicating reliable communication even under attack conditions. Energy consumption was efficiently managed, with slight variations observed across datasets due to differing network conditions. Latency was maintained consistently within acceptable limits, with an average range of 115 to 125 ms. The mitigation efficiency results highlight the model’s ability to counter attacks effectively, with efficiency rates greater than 85%. The detailed comparison between datasets and metrics is shown in Figure 7, Figure 8, Figure 9, Figure 10 and Figure 11, highlighting the adaptability and robustness of the model against various cyberattacks.

### 6.3. Numerical Analysis of the Proposed System

To validate the effectiveness of the proposed reinforcement learning-based adaptive encryption framework, a numerical analysis was performed based on key security and performance metrics. The system was evaluated in different cyberattack scenarios, including DDoS, data injection, black-hole, wormhole, and selective forwarding attacks. The objective of this analysis is to quantify the capability of the model in detecting and mitigating attacks while ensuring energy-efficient encryption. The numerical evaluation considers parameters such as attack request rates, entropy deviations, packet drop rates, and transmission delays. By applying the mathematical formulations presented in the security analysis, the probability of attack detection and the overall security effectiveness of the proposed model are calculated. The results provide insights into how the reinforcement learning framework dynamically adjusts encryption levels to mitigate cyber threats while maintaining optimal network performance.

To ensure the analysis closely reflects real-world WSN conditions, appropriate values were selected based on prior research and practical deployment considerations. The DDoS attack request rate was assumed to be $\lambda_{a}=20$ packets per second, reflecting the moderate attack scenarios commonly observed in IoT and WSN environments, where attack traffic typically ranges between 15–25 packets per second. The threshold for anomaly detection was set at $\lambda_{\mathrm{thresh}}=15$, representing a normal network traffic limit. For data injection attacks, entropy values were chosen based on information entropy analysis in wireless networks. Normal sensor readings exhibit relatively low entropy, with an expected value of $H_{\mathrm{expected}}=0.85$, while manipulated data often show a significant deviation, reaching H(X)=1.2. If the entropy deviation exceeds a predefined threshold of τ=0.3, the system classifies the event as an attack.

The packet drop rates for black-hole and selective forwarding attacks were determined on the basis of typical attack behaviors in WSNs. In black-hole attacks, malicious nodes drop approximately 20–30% of packets, leading to an assumed value of $D_{\mathrm{drop}}=2500$ packets dropped out of $D_{\mathrm{sent}}=10000$ total transmissions. Selective forwarding attacks specifically target critical packets, with an estimated drop rate of 15%, corresponding to $D_{\mathrm{critical}}=1500$ dropped packets. Transmission delay values were chosen to reflect normal and attack-induced delays. Under normal conditions, WSN transmissions experience an average delay of $T_{\mathrm{expected}}=2.5$ ms, whereas wormhole attacks introduce substantial delays due to malicious rerouting, leading to an observed delay of $T_{\mathrm{delay}}=6.0$ ms. The assumed mitigation success rates for each type of attack were derived from previous studies on intrusion detection and adaptive security mechanisms in WSNs, ensuring that detection probabilities are realistic and achievable.

**DDoS Attack Detection Probability:** In a DDoS attack, the adversary floods the network with excessive traffic. The detection probability is modeled as presented in Equation (21).

(21) $$P_{\mathrm{DDoS}\;\mathrm{Detect}}=\frac{\lambda_{a}}{\lambda_{a}+\lambda_{\mathrm{thresh}}}$$

where $\lambda_{a}=20$ → attack request rate (packets per second); $\lambda_{\mathrm{thresh}}=15$ → threshold for detecting abnormal packet rates as given in Equation (22).

(22) $$P_{\mathrm{DDoS}\;\mathrm{Detect}}=\frac{20}{20+15}=\frac{20}{35}=0.571$$

Thus, the proposed system detects a DDoS attack with 57.1% probability.

**Data Injection Attack Detection:** A data injection attack alters or injects false sensor data. This is detected based on entropy deviation given in Equation (23).

(23) $$\Delta H=|H\left(X\right)-H_{\mathrm{expected}}|$$

where $H_{\mathrm{expected}}=0.85$ gives the expected entropy under normal conditions, H(X)=1.2 gives the observed entropy in the attack scenario, and τ=0.3, which is the threshold for the detection of entropy deviation, given as ΔH=|1.2−0.85|=0.35>0.3. Since ΔH>τ, the attack is successfully detected. Thus, $P_{\mathrm{Data}\;\mathrm{Injection}\;\mathrm{Detect}}=1$ indicates a 100% success rate in detecting data injection attacks.

**Black-Hole Attack Probability:** A black-hole attack occurs when a malicious node drops all incoming packets instead of forwarding them. The probability of attack can be calculated using Equation (24).

(24) $$P_{\mathrm{Blackhole}}=\frac{D_{\mathrm{drop}}}{D_{\mathrm{sent}}}$$

where $D_{\mathrm{drop}}=2500$ gives the packets dropped by the black-hole node and $D_{\mathrm{sent}}=10000$ is the total packets sent in the network. Hence, $P_{\mathrm{Blackhole}}=\frac{2500}{10000}=0.25$. Thus, 25% of the packets are affected by the black-hole attack and the system needs mitigation.

**Wormhole Attack Probability:** A wormhole attack creates a shortcut tunnel between two adversary-controlled nodes to alter routing. The attack probability is modeled as Equation (25):

(25) $$P_{\mathrm{Wormhole}}=\frac{T_{\mathrm{delay}}}{T_{\mathrm{expected}}}$$

where $T_{\mathrm{expected}}=2.5$ ms → expected transmission delay and $T_{\mathrm{delay}}=6.0$ ms → observed transmission delay in wormhole attack. Hence, $P_{\mathrm{Wormhole}}=\frac{6.0}{2.5}=2.4$. This value being greater than one indicates a significant delay anomaly, confirming a wormhole attack is highly likely.

**Selective Forwarding Attack Probability:** In a selective forwarding attack, the adversary selectively drops certain packets. The probability of attack is given in Equation (26).

(26) $$P_{\mathrm{Selective}\;\mathrm{Forwarding}}=\frac{D_{\mathrm{critical}}}{D_{\mathrm{total}}}$$

where $D_{\mathrm{critical}}=1500$ → number of dropped critical packets and $D_{\mathrm{total}}=10000$ → total transmitted packets. It is calculated as $P_{\mathrm{Selective}\;\mathrm{Forwarding}}=\frac{1500}{10000}=0.15$. Thus, 15% of critical packets are affected, which requires mitigation based on encryption.

**Overall Security Effectiveness (%):** The proposed system mitigates threats dynamically, with the following mitigation success rates as presented in Table 6.

The overall security effectiveness of the proposed model is computed using Equation (27),

(27) $$\begin{array}{cc} SE & =\left(M_{\mathrm{DDoS}}\cdot P_{\mathrm{DDoS}\;\mathrm{Detect}}+M_{\mathrm{Data}\;\mathrm{Injection}}\cdot P_{\mathrm{Data}\;\mathrm{Injection}\;\mathrm{Detect}}\right)+\\ \; & \;\left(M_{\mathrm{Blackhole}}\cdot P_{\mathrm{Blackhole}}+M_{\mathrm{Wormhole}}\cdot P_{\mathrm{Wormhole}}\right)+\\ \; & \;\left(M_{\mathrm{Selective}\;\mathrm{Forwarding}}\cdot P_{\mathrm{Selective}\;\mathrm{Forwarding}}\right)\times 100 \end{array}$$

Substituting values, we have SE=(0.95×0.571)+(0.92×1)+(0.94×0.25)+(0.90×2.4)+(0.91×0.15) and, further, SE=0.542+0.92+0.235+2.16+0.136; hence, SE=3.993×100=399.3%. Since the maximum possible security effectiveness is 100%, the value is normalized as $SE_{\mathrm{normalized}}=\frac{3.993}{5}\times 100=79.86\%$. Thus, the proposed system achieves an overall security effectiveness of 79.86%, successfully mitigating threats while optimizing encryption usage.

**The overall latency analysis:** The latency findings presented in Figure 10 were analyzed by measuring packet transmission time across different datasets, including AWID, IoT-23, and WSN-BFSF, under various cyberattack scenarios. A detailed statistical evaluation was performed to assess the consistency of these latency values using mean, standard deviation, and confidence intervals. The mean latency values observed for the AWID, IoT-23 and WSN-BFSF datasets were 118, 120, 123, and 125 ms, respectively. The standard deviations for these datasets were 3.2, 4.1, 5.6, and 6.8 ms, respectively, indicating higher latency variations in the IoT-23 and WSN-BFSF datasets. A higher standard deviation in the WSN-BFSF dataset suggests increased fluctuations due to cyberattacks, particularly selective forwarding and wormhole attacks, which significantly impact packet transmission.

To evaluate the reliability of the latency values, a 95% confidence interval was computed. The confidence interval for the AWID dataset was [115.6 ms, 120.4 ms], indicating high stability in latency performance. Similarly, the IoT-23 dataset had a confidence interval of [116.3 ms, 123.7 ms]. The WSN-BFSF dataset, which had the highest latency fluctuations, recorded a confidence interval of [118.6 ms, 131.4 ms]. These results confirm that black-hole and wormhole attacks significantly impacted latency variations in the 613 and WSN-BFSF datasets, leading to increased packet retransmissions and congestion.

Furthermore, the proposed model exhibited a latency reduction of 12.5% compared to traditional routing approaches, ensuring stable transmission across all datasets. The results validate that the security measures integrated into the model effectively mitigate the latency impact caused by various cyberattacks. The confidence interval analysis further reinforces the reliability of the latency values, with minimal overlap between datasets, indicating distinct latency behaviors under different attack conditions.

The numerical analysis validates the proposed reinforcement learning-based encryption model, demonstrating high detection accuracy and efficient cyber threat mitigation. The model effectively detects DDoS attacks with a probability of 57.1%, ensures a 100% detection rate for data injection attacks through anomaly monitoring based on entropy, and mitigates black-hole attacks, where 25% of packets are dropped. Furthermore, wormhole attacks are identified based on a significant transmission delay anomaly (2.4× expected delay), and selective forwarding attacks affect 15% of critical packets. The overall security effectiveness of the proposed model is calculated as 79.86%, which confirms its ability to dynamically optimize encryption strength while conserving energy. The findings establish that reinforcement learning-driven adaptive encryption enhances cyber threat resilience, ensuring robust security while maintaining network efficiency in real-time WSN deployments.

## 7. Conclusions

This research demonstrates the feasibility and effectiveness of reinforcement learning, particularly Q-learning, in enabling adaptive encryption for cyberattack mitigation in WSNs, paving the way for secure and efficient next-generation wireless networks. This paper proposed a novel reinforcement learning-based adaptive encryption framework that leverages Q-learning to dynamically adjust encryption levels in response to real-time network conditions and threat levels. The proposed framework addresses key limitations of traditional and static encryption techniques by incorporating a reward-driven approach to optimize the trade-off between security, energy consumption, and communication latency. By evaluating the model against various cyberattack scenarios, including DDoS, black-hole, and data injection attacks, the framework demonstrated superior performance in detection accuracy, PDR, energy efficiency, and mitigation effectiveness. The results further highlighted the potential of Q-learning to improve security adaptability while maintaining critical network performance metrics. In addition to dynamic encryption, the integration of anomaly detection mechanisms ensured early identification of threats, allowing proactive responses to evolving attacks. Comparisons with existing techniques revealed that the proposed framework significantly reduces energy consumption and latency while providing robust security against both known and novel threats. This makes it a viable solution for real-time applications in resource-constrained WSN environments.

Future research will focus on enhancing the reinforcement Q-learning-based adaptive encryption model by extending its applicability to large-scale WSN and IoT ecosystems. One key direction is the deployment of the model in real-world WSN environments, integrating heterogeneous sensor nodes to evaluate performance under practical energy, latency, and security constraints. Additionally, multi-agent reinforcement learning will be explored to enable decentralized encryption decision-making, reducing reliance on centralized control mechanisms. To further improve security, blockchain-based key management will be investigated for ensuring integrity in adaptive encryption policies, preventing key compromises in adversarial settings. The model’s ability to defend against adversarial reinforcement learning attacks will be strengthened by incorporating adversarial training techniques and anomaly-aware Q-learning mechanisms. Another important direction could be the introduction of an adaptive hyperparameter tuning mechanism using meta-reinforcement learning to dynamically adjust Q-learning parameters based on network conditions, further enhancing learning efficiency and convergence speed in highly dynamic WSN environments.

## Figures and Tables

**Table 1:** Comparison of previous studies on WSN security and encryption mechanisms.

Ref.	Methodology	Advantages	Limitations	Results
Mohaned Anwar et al. [17] (2024)	Blockchain-based encryption with dynamic key generation	Improved security and throughput	High computational cost	Latency reduced by 15%, accuracy 91%
Arivumani et al. [18] (2024)	Convolutional LSTM for intrusion detection	High detection accuracy	Requires large training data	98% precision, few false positives
Wang et al. [19] (2024)	Deep reinforcement learning for trust model in underwater WSNs	Mitigates malicious node attacks	Requires online learning updates	95% attack detection accuracy, energy-efficient routing
Rasool et al. [20] (2025)	Hybrid optimization for WSN security	Efficient threat detection	High computational complexity	96% threat detection rate, moderate energy efficiency
Saveetha et al. [21] (2024)	Federated learning with blockchain for DDoS mitigation	Decentralized security	High resource consumption	92% accuracy, reduced latency
Rehman et al. [14] (2022)	Blockchain-enhanced reinforcement learning	Secure distributed encryption	Increased computation overhead	93% anomaly detection accuracy, low latency overhead
Fascista et al. [15] (2022)	Adaptive encryption with real-time feedback mechanism	Optimized energy usage	Limited scalability in large networks	90% improvement in network resilience, energy savings
Han et al. [30] (2024)	Trust-aware clustering algorithm for IoT networks	Enhances data security	Higher energy consumption	88% security effectiveness, 20% reduced overhead
Saleh et al. [31] (2024)	Blockchain and machine learning integration for security	Improved anomaly detection	High storage and processing requirements	97% anomaly detection accuracy, enhanced privacy protection
Singh et al. [32] (2024)	Hybrid SVM and RF model for DDoS detection in SDN	High detection accuracy	Model complexity increases processing time	94% detection rate, improved network resilience
Rama lakshmi et al. [29] (2024)	Distributed multi-controller approach for mitigating DDoS in fog computing	Scalability in large networks	Requires extensive resource allocation	90% mitigation efficiency, reduced latency
Altaweel et al. [8] (2024)	Security strategies for opportunistic mobile networks	Detects various attacks like black-hole and wormhole	High false positive rates in anomaly detection	91% threat classification accuracy, moderate latency

**Table 2:** List of abbreviations and acronyms used in equations and algorithms.

Acronym	Definition	Acronym	Definition
Q(s,a)	Q-value for state *s* and action *a*	α	Learning rate in Q-learning
γ	Discount factor in reinforcement learning	R(s,a)	Reward function for state *s* and action *a*
π(s)	Policy function mapping state *s* to action probabilities	V(s)	Value function of state *s*
ϵ	Exploration–exploitation parameter in ϵ-greedy strategy	δ	Temporal Difference (TD) error
θ	Model parameters in deep reinforcement learning (DRL)	*∇*	Gradient operator used in optimization
E[·]	Expectation operator	τ	Target update parameter in Deep Q-Networks (DQNs)
λ	Eligibility trace decay factor in TD learning	σ	Standard deviation in Gaussian exploration
L	Loss function for policy optimization	$P(s^{\prime }|s,a)$	State transition probability from *s* to $s^{\prime }$ given action *a*
A(s,a)	Advantage function in Actor–Critic methods	β	Entropy regularization parameter

**Table 3:** Dataset parameters for experimental setup.

Parameter	Description
Number of Sensor Nodes	50 sensor nodes with distinct energy levels and sensing capabilities
Threat Levels	Low, medium, and high threat levels simulated as random attack intervals
Energy Levels	Uniformly distributed between 10J and 100J
Simulation Duration	Fixed period of 300 s during which nodes adaptively transmitted data

**Table 4:** Performance metrics comparison of proposed and baseline models.

Model	Energy Consumption (J)	PDR (%)	Latency (ms)	Security Effectiveness (Threats Detected)
AES-128 Fixed	72.5	80.1	190	6
Lightweight Block Cipher	55.0	84.3	160	8
Elliptic Curve Cryptography	65.2	86.7	175	7
Hybrid AES-RSA	70.4	88.5	150	10
Dynamic Threshold Encryption	52.8	89.9	140	12
Blockchain-Based Lightweight Encryption	60.7	88.9	135	11
Proposed Model	45.2	92.5	120	15

**Table 5:** Performance metrics across cyberattacks for different datasets.

Metric	Dataset	DDoS	Data Injection	Black-Hole	Worm Hole	Select Forward
Accuracy (%)	AWID Dataset	98	97	97	96	96
	IoT-23 Dataset	98	96	97	94	96
	WSN-BFSF Dataset	97	95	95	93	95
PDR (%)	AWID Dataset	95	94	94	93	93
	IoT-23 Dataset	95	92	93	91	92
	WSN-BFSF Dataset	93	91	92	91	92
Energy (J)	AWID Dataset	73	70	71	69	70
	IoT-23 Dataset	73	70	71	68	69
	WSN-BFSF Dataset	74	70	71	69	70
Latency (ms)	AWID Dataset	120	115	118	122	116
	IoT-23 Dataset	122	116	119	124	117
	WSN-BFSF Dataset	125	118	121	126	119
Mitigation (%)	AWID Dataset	91	89	90	87	88
	IoT-23 Dataset	90	88	89	86	87
	WSN-BFSF Dataset	89	87	88	85	86

**Table 6:** Mitigation success rates of proposed model.

Attack Type	Mitigation Success Rate
DDoS	95%
Data Injection	92%
Black-Hole	94%
Wormhole	90%
Selective Forwarding	91%

## Data Availability

Data are contained within the article.
